# ACTonHEALTH study protocol: promoting psychological flexibility with activity tracker and mHealth tools to foster healthful lifestyle for obesity and other chronic health conditions

**DOI:** 10.1186/s13063-018-2968-x

**Published:** 2018-11-29

**Authors:** Roberto Cattivelli, Gianluca Castelnuovo, Alessandro Musetti, Giorgia Varallo, Chiara A. M. Spatola, Francesco Vailati Riboni, Anna Guerrini Usubini, Fabio Tosolin, Gian Mauro Manzoni, Paolo Capodaglio, Alessandro Rossi, Giada Pietrabissa, Enrico Molinari

**Affiliations:** 1 Istituto Auxologico Italiano IRCCS, Psychology Research Laboratory, Verbania, Italy; 2 Department of Psychology, Catholic University of Milan, Milan, Italy; 3 Department of Humanities, Social Sciences and Cultural Industries, University of Parma, Parma, Italy; 4 Faculty of Psychology, eCampus University, Novedrate, Italy; 5 AARBA, Association for the Advancement of Radical Behavior Analysis, Milan, Italy; 6 Interdepartmental Center for Family Research, Department of Philosophy, Sociology, Education, and Applied Psychology, University of Padova, Padova, Italy; 7 Istituto Auxologico Italiano IRCCS, Rehabilitation Unit and Research Laboratory of Biomechanics and Rehabilitation, S Giuseppe, Piancavallo, Oggebbio Verbania, Italy

**Keywords:** Acceptance and Commitment Therapy - ACT, Wearable, Activity trackers, Health promotion, Obesity, Chronic health condition, Behavior modification, Psychological flexibility, Randomized clinical trial, Multiple-baseline single-subject design

## Abstract

**Background:**

Obesity and the state of being overweight are increasing steadily and becoming a global epidemic. Recent research reports 64% of the adult population as overweight in Europe and the USA. The social and economic impacts are increasing, and most of the rehabilitation programs, while effective in the short term, do not produce long-lasting results. An explanatory model from a behavioral perspective can describe the phenomena with the lack of sources of reinforcement related to healthful habits in a daily life context.

**Methods/design:**

A randomized clinical trial combining single-subject studies and a four-arm group design will be conducted to compare the effect of the current standard in obesity treatment to Acceptance and Commitment Therapy (ACT) and wearable technology at different times, before starting intervention, at the end, and at follow-up visits of 3, 6, and 12 months measuring changes over time of physical activity and psychological well-being.

**Discussion:**

The goal of this project, combining ACT and wearable technology, is to develop an effective intervention, efficient and sustainable, which even after discharge can provide adequate contingencies of reinforcement in the natural environment, integrating systematic measurements, continuous feedback, and individualized, values-based objectives. The intervention is aimed to provide a contingent reinforcement for healthful behaviors instead of reinforcing only the achievement of a significant weight loss.

The aim of the project, combining Acceptance and Commitment Therapy and Wearable Technology, is to develop an effective, efficient and sustainable intervention able to provide a contingent reinforcement for healthy behaviors. The intervention is aimed to promote adequate healthy behaviors in the natural environment, integrating systematic measurements, continuous feedback and individualized values-based objectives, instead of reinforcing only the achievement of a significant weight loss.

**Trial registration:**

ClinicalTrials.gov, NCT03351712. Registered on 24 November 2017.

**Electronic supplementary material:**

The online version of this article (10.1186/s13063-018-2968-x) contains supplementary material, which is available to authorized users.

## Background

The condition of being overweight is an increasing problem worldwide and is becoming an epidemic both in Europe and the USA. According to recent estimates, more than 60% of adults are overweight, and obesity affects around 35% of the population [59]. In the USA, the healthcare system, in order to cope with obesity and related diseases, spends the sum of $100 billion [24, 58, 84], and in Europe costs are currently similar [84] with a rising percentage [44]. Health risks related to being overweight include psychological difficulties, depression and stigma, physical problems and heart diseases, cancer, and respiratory diseases, not to mention musculoskeletal and metabolic problems [22, 23, 29, 41, 42, 55, 58, 75, 82].

Obesity is a chronic health condition [15], but it is also a major risk factor for various diseases, both chronic and acute [74]. This problem is currently rising, with prevalence data and epidemiological estimates that should be a matter of great concern [60]. Despite strong biological components and hereditary aspects, obesity is primarily linked to incorrect habits of everyday life, mainly eating and physical activity habits [16, 63]. Numerous studies [29, 45, 59] have documented that regular physical activity and well-informed choices in food intake can prevent obesity and related conditions. The main challenge in addressing obesity and associated disorders is developing and providing sustainable comprehensive programs that include combinations of physical activity, dietary aspects, and psychological interventions [17–19]. Interventions developed in this way, including dietary education protocols, physical rehabilitation and exercise programs, and nutritional, endocrinal, psychological, surgical, and pharmacological treatments, are generally effective in the short term, despite many of them being characterized by high costs and long periods of hospitalization [58, 84, 89]. An analysis of the literature [16, 24, 56] suggests the superiority of intensive multidisciplinary interventions: dietary programs and psychological and physical rehabilitation. One of the problems of such interventions is that they are more easily implementable during a hospitalization period, with high costs associated. However, despite good evidence of effectiveness, the long-term results are generally limited [27, 46]. In general, availability, costs, compliance, and long-term efficacy are important limitations to this variety of approaches [4, 11, 15, 20, 25, 64]. Frequently, obese individuals will regain about 30% of the weight lost within the first year [28]. Often the inpatients return to pre-treatment weight after only 3 years [16]. Psychological interventions are generally assumed to be one of the core pillars in obesity treatment, supporting lifestyle change including dieting and physical activity. Among others, cognitive behavioral therapy (CBT) interventions are generally considered the gold standard for addressing “globesity” [5, 15]. In particular, treatments aimed to promote a healthful lifestyle, with a strong focus on fostering physical activity, have proven efficacy [43, 54, 91] and are generally considered the gold standard in the field [34, 39, 40, 72, 90]. CBT-based programs show generally good outcomes; nevertheless, according to recent research, the outcomes of these programs usually do not last in the long term [19, 40, 74, 80]. In order to maintain outcomes in a long-term perspective, recently *acceptance and commitment therapy (ACT)* has been considered. Both as an add-on treatment and in a combined format, ACT could be beneficial specifically for improving long-term weight loss outcomes. Recent developments in mindfulness and acceptance-based interventions provide a potential avenue for treatment development [22].

### The present study

Principal reasons for the long-term failure of obesity interventions are the lack of long-term sources of reinforcement for healthful habits acquired during the intensive treatment period [14, 31, 32]. The tradition of behavioral sciences has developed models of interpretation, explanation, and pragmatic operation supported by evidence of effectiveness and efficiency in the promotion of desired behaviors in various contexts, for example, smoke cessation [10, 81], pathological Internet use [69, 70], the ability to live with chronic diseases [6, 48, 66], and the promotion of safety behaviors in working environments [71, 88]. For this reason, behavior analysis in general, and in particular *contextual behavioral science*, can play a decisive role in the prevention of obesity and in the maintenance of the results achieved within short intensive protocols, usually held in hospitals. By modifying deeply rooted and long-term acquired habits, obese patients can obtain more psychological and physical health, reduce risk factors, and improve their overall health, wellness, and quality of life [53, 59] with benefits not only for the individual but also for his social environment. During rehabilitation patients affected by obesity are frequently in contact with contingencies extremely different from those occurring in their daily lives. During rehabilitation or hospitalization, contingencies are efficiently shaped to promote desired behaviors and decrease actions that are identified as dysfunctional, with both aversive and reinforcing contingencies. Later on, after the treatment, in the patient’s natural environment initially behaviors are maintained, but since they were reinforced in an environment that provides adequate contingencies, they often undergo a process of extinction consequently with cessation of reinforcement [79]. At the same time, outside the hospital, dysfunctional behaviors, such as avoiding physical activity and eating tasty food, are reinforced in a systematic and contingent way. This tendency to extinction of functional and healthful habits and reinforcement of dysfunctional habits leads, in the long term, to gaining of the weight lost laboriously, which also leads to lower levels of well-being [77]. Although the existing programs already provide protocol phases to be implemented once patients are home, often the patients fail to adhere to nutrition programs and planned activities. On the contrary, they return frequently to previous habits and show a high dropout rate at follow-ups [76].

To overcome the limited long-term adherence after the hospitalization period, it is necessary to find a way to also give significant, contingent, and informative feedback after the rehabilitation phase. In this regard the use of activity trackers represents a clear, valid, and “smart” use of new technologies, linked to well-established behavioral science [78]. Regular physical activity is associated with numerous psychological and physical benefits. Physical activity maintenance over the long term is even more difficult to achieve, with a lead role assigned to motivation that is reported as an important factor for adherence to the initial decision to change the behavior as well as for actually maintaining the behavioral change. Smartphone technology provides an opportunity to deliver physical activity interventions remotely. Several studies have demonstrated the validity of the Fitbit activity monitor [1]. A “smart” physical activity application could track physical activity in real time using the built-in accelerometer, provide instant feedback on steps taken or calories burned, and deliver immediate reinforcement of physical activity. This application could also track progress toward physical activity goals over time and include other evidence-based features enabling goal setting and problem solving. Activity trackers could be used by healthcare workers and researchers to observe daily activities and make recommendations based on short- and long-term trends in behavior. These electronic devices could help consumers to monitor their health even outside the hospital. Therefore, newer activity trackers, such as the Fitbit, could be utilized in these types of intervention to facilitate self-monitoring and goal setting, which are essential for initiating and maintaining physical activity in the long term [12]. Fitbit provides a detailed but user-friendly report of physical activity data utilizing a color-coded scheme on the screen (for example, showing different colors of the activities for achieving daily goals). In addition, other features, such as smiley faces that appear when daily goals are achieved and badges awarded for specific accomplishments (for example, reaching a distance or step-count milestone), could be reinforcing and motivating for participants.

Perfectly incorporating the use of technology to provide meaningful feedback, treatments based on third-wave intervention, such as ACT, or, more generally, acceptance and mindfulness-based approaches are promising to support long-lasting behavioral change in the field of healthful habits promotion [42, 46, 60, 61]. Mindfulness and acceptance-based interventions, often defined as third-generation behavioral approaches, have as their objective the modification of one’s relationship to undesirable thoughts, feelings, or bodily sensations ([3, 13, 21, 22, 49, 50, 83]). Recently, third-generation interventions have rapidly gained popularity. ACT, one of the new wave’s most increasingly used interventions, is empirically supported for a wide variety of psychological and behavioral problems, including chronic pain, anxiety, depression, and smoking cessation [3, 13, 21, 22, 49, 50, 83]. ACT uses three cardinal processes (acceptance, mindfulness, and values) to promote psychological flexibility, definable as the ability to pursue values-based action even in the presence of unwanted thoughts, feelings, and bodily sensations. In the specific context of weight control, ACT aims to encourage healthful behavioral patterns in accordance with stated values, while providing mindfulness and acceptance skills to enhance commitment to values-based behavior [7, 61].

### Hypothesis, goals, and expected results

The project aims to promote a long-term change in lifestyle as part of a multidisciplinary intervention, in close collaboration with physicians, the service of clinical nutrition, and osteoarticular rehabilitation.

The main goal of this study is test the efficacy of combining behavioral change technologies through contingent and meaningful feedback provided by wearable devices with an intervention based on ACT, a well-studied psychological treatment developed in a contextual behavioral framework. The combinations of this approach should be feasible and efficient, compared with the gold standard treatment, and have lower costs. Four experimental conditions are provided: (1) usual care during hospitalization with programmed follow-up checks, (2) contingent feedback on daily life activities through activity trackers or stand-alone wearable devices, (3) stand-alone ACT-based intervention, (4) a combination of the previous two conditions: ACT-based intervention and activity tracker with feedback.

The primary outcome, or dependent variable, of the study is level of physical activity as collected by wearable electronic devices, a largely used parameter in scientific investigations [33, 68]. Other outcomes—for example, time of sleep—are collected by wearables, and others are provided by self-report through the web platform. The independent variable of the study is the type of treatment (ACT intervention and feedback provided by activity trackers). For the purpose of the study we collect data about daily steps and a workout log for each participant.

The intervention aims to help people in their everyday environment to more consistently keep the choices they have made and follow appropriate recommendations on diet and movement. The project is directed to participants in the multidisciplinary rehabilitation program at St. Joseph Hospital - Verbania, Istituto Auxologico Italiano, a private research institute that also works for the Italian national health system. The recruitment will take place progressively, and subjects will be randomized to the four experimental conditions set by the protocol. Data will be analyzed in different experimental single-subject over multiple-baseline designs [2, 8]. The subjects are also grouped according to the experimental conditions to allow statistical analysis of experimental intergroup designs, consistent with the approach of a randomized controlled trial (RCT).

**Fig. 1:**
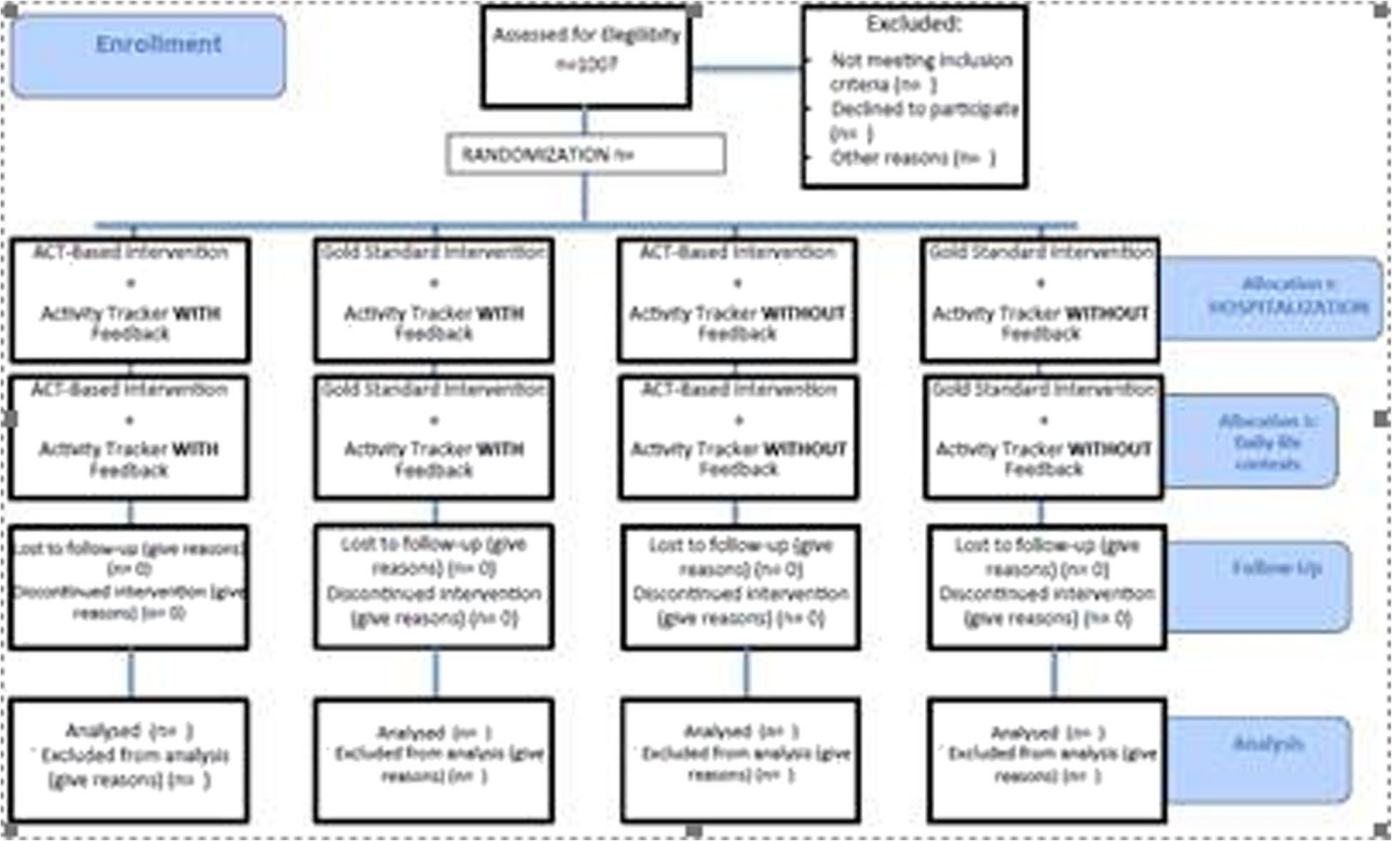
CONSORT flow diagram

**Fig. 2:**
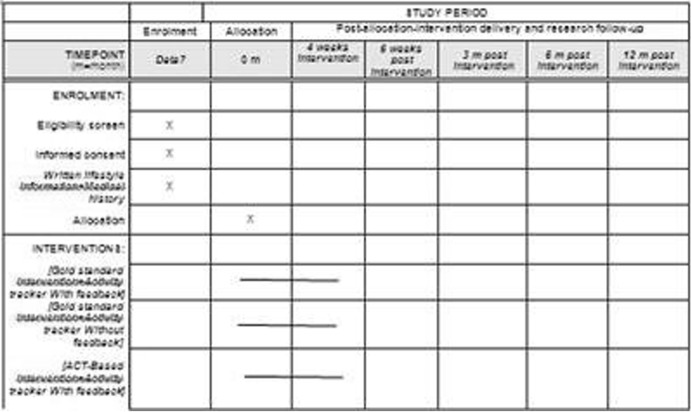
SPIRIT figure

**Fig. 3:**
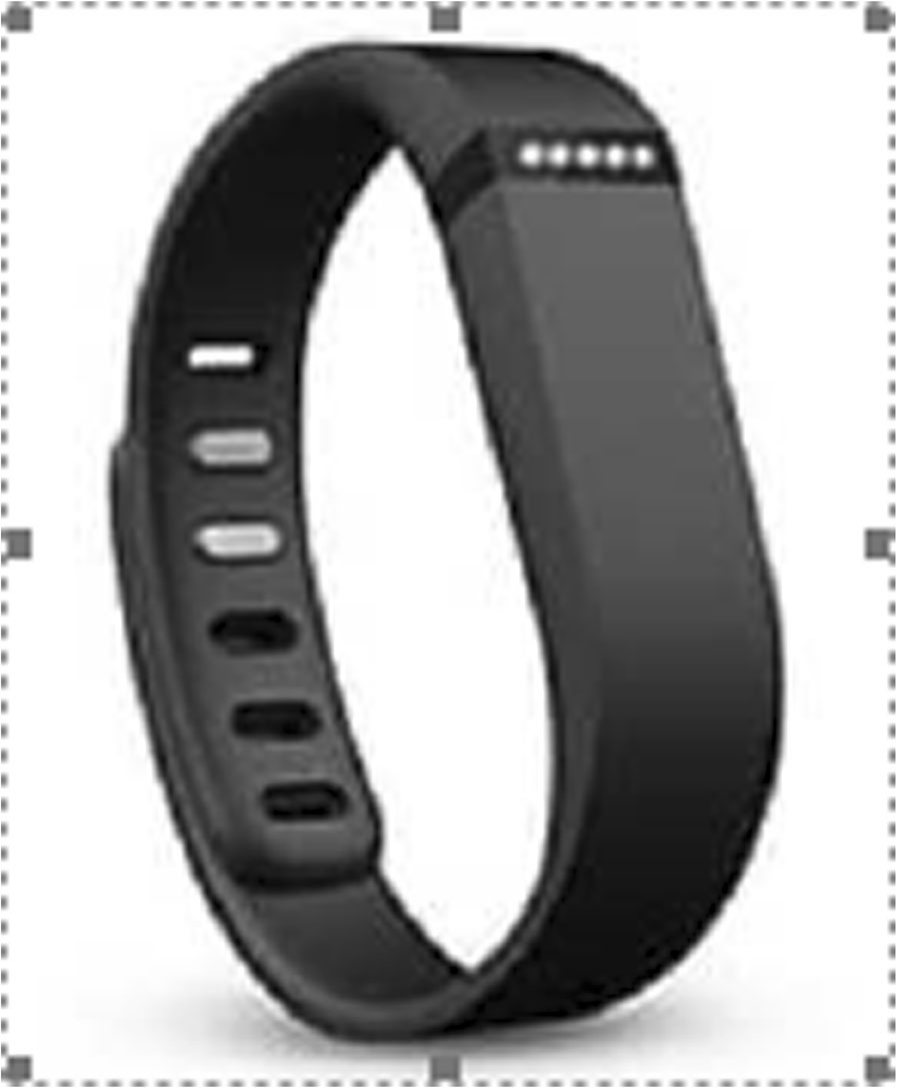
Wearable sample

The CONSORT diagram of the study is shown in Fig. 1, and the SPIRIT schedule for this trial is given in Fig. 2, and a sample of wearable device is shown in Fig. 3. Table 1 illustrate the experimental conditions, and Additional files 1, 2 and 3 are provided, consisting respectively in the complete SPIRIT checklist for the study, the Consort Full Checklist and the TIDieR checklist.

**Table 1:** Experimental conditions

	Brief ACT intervention	Medical rehabilitation, motivational support, and psycho-education
Contingent and informative feedback (SR+) provided by activity tracker	ACT + + Feedback provided by activity tracker	No ACT – traditional motivational intervention + feedback provided by activity tracker
Non-contingent and episodic feedback (SR+) – No feedback provided by activity tracker, usual post-rehabilitation information	ACT without feedback provided by activity tracker	Traditional medical rehabilitation, motivational support, and psycho-education without feedback provided by activity tracker

SR+ states for Positive Reinforcement

## Methods

### Study design

Recently the Oxford Centre for Evidence-Based Medicine (http://www.cebm.net/) has established that the level of scientific evidence of single-subject experimental designs (when some conditions are respected) has a rank that is similar to that of systematic reviews and just below that of a meta-analysis. Additionally the single-subject design is considered to have a higher ranking than the more conventional intergroup design often used for randomized clinical trials [51]. In the present study we integrate an experimental single-subject design that is repeated in multiple baselines. For the study an ample sample is available, and a significant number of data points is taken into account. This is made possible by the use of information technology, with activity trackers/wearables, which allow individual, continuous, and constant data collection.

This data analysis strategy is being performed with the intention-to-treat (ITT) method. This specific choice was adopted with two main purposes: maintaining similar treatment groups apart from randomization, and allowing non-compliance and deviations from the clinicians and researcher’s policy, keeping most of the possible exceptions under control. The continuous sampling and data collection, made possible mainly by a technological system of automatic devices, makes the use of a large sample of subjects possible. Moreover, it allows an experimental analysis of the participants even with the methodology of the comparison between randomized groups. For the side of group analysis this study is a small size RCT, with four different experimental conditions, depending on two different independent variables, each on two levels (present or absent), with a classic 2 × 2 design factorial. For every condition all the participants of the study follow the same inpatient, 4-week rehabilitation program followed by a 16-week outpatient phase. Psychometric and direct measures are collected during both the inpatient and outpatient phases, and at follow-up at 3, 6, and 12 months.

The experimental conditions are as follows:

- Gold Standard Intervention plus Activity Tracker WITHOUT Feedback
- Gold Standard Intervention plus Activity Tracker WITH Feedback
- ACT-Based Intervention plus Activity Tracker WITHOUT Feedback
- ACT-Based Intervention plus Activity Tracker WITH Feedback

### Gold Standard Intervention plus Activity Tracker WITHOUT Feedback

#### Medical rehabilitation, motivational support, and psycho-education

During the inpatient phase, participants will participate in the intensive 4-week hospital-based and medically managed rehabilitation program for weight reduction. All patients will be placed on a hypocaloric nutritionally balanced diet tailored to the individual after consultation with a dietitian. Furthermore, they will receive nutritional counseling provided by dietitians, physical activity training provided by physiotherapists, and motivational support with elements of psycho-education provided by physicians trained and informed by psychologists-psychotherapists.

The nutritional rehabilitation program aims to promote change in eating habits and consists of both individual and group sessions twice a week and includes information on obesity and related health risks, setting of realistic goals for weight loss, healthful eating in general, general nutrition and core food groups, weight management, and strategies for preventing relapse. The individual dietary counseling involves evaluation of nutrient intake and adequacy, nutritional and anthropometric status, dietary assessment, eating patterns, history of being overweight, and readiness to adopt change. Another meeting will be scheduled to deliver the activity tracker and linked app. The trackers are presented as mere data collection devices, for medical use only, that do not provide any type of feedback or data to the participants.

### Gold Standard Intervention plus Activity Tracker WITH Feedback

#### Behavioral change condition with activity tracker

In this experimental condition, participants will be provided the same rehabilitation program for the 4-week inpatient phase as in the control condition. In addition, for these subjects we will implement a stepped protocol using wearable devices/activity trackers to collect information about daily physical activity and providing meaningful and informative feedback. The additional procedure starts during the inpatient phase with delivery and explanation of the use of the wearable devices. In this meeting, longer than the one described for the control condition, experimenters provide information, set individualized goals, and explain the feedback to be delivered by the electronic wearable devices after the end of the inpatient phase. All subjects allocated to this condition will be clustered by date of entrance in the program; then, for each cluster, the length of the baseline phase will be randomly assigned. According to these allocations, the wearable device will start to provide feedback at different times for each participant in the cluster, to directly check the effect of contingent and informative feedback, one of the two independent variables of the study, on the daily level of physical activity, our primary outcome and dependent variable. The stepped protocol includes automatically generated feedback for achieving individualized goals in physical activity or related healthful habits. If the goal is missed one day, a daily report is automatically sent, but if any participant misses repeatedly, the individualized goal researchers provide an individualized contact by text message or e-mail.

### ACT-Based Intervention plus Activity Tracker WITHOUT Feedback

#### ACT-based intervention

In this experimental condition, the subjects followe the normal medical rehabilitation program described above for the first experimental condition. In addition, within the 4-week period of the inpatient phase, we provide a brief ACT intervention during the hospitalization, followed by consulting/therapy sessions provided by a combination of phone calls, e-mails, and text messages in the outpatient phase. The total duration of one-to-one interventions during the hospitalization is 3 h, with four 45-min sessions during the rehabilitation. For the outpatient phase the ACT intervention includes a monthly 30 min Skype-telephone session, starting after the end of hospitalization, that includes weekly text messages or e-mails but is not linked with any kind of data regarding participants of the study.

The ACT-based interventions include the following: (1) acceptance or “willingness,” which involves the active awareness of difficult private experiences without unnecessary and often dysfunctional attempts to control or avoid unpleasant emotions. “Acceptance” is the alternative to emotional and experiential avoidance. Participants will be trained to improve adherence to a healthful lifestyle by increasing willingness to experience unwanted and difficult sensations and urges while engaging in health-related behaviors. (2) Mindfulness is definable as the process by which we can engage in the present moment, adopting an open and curious attitude. Additionally, mindfulness aims to increase self-awareness by noticing all internal experiences, with a non-judging stance, becoming aware of judgments and being able to let them go. Mindfulness allows one to explore previous control-based attempts to cope with unpleasant thoughts, emotions, and sensations and to demonstrate the ineffectiveness of control while pursuing a meaningful life. (3) With the “defusion” process, participants will be encouraged to alleviate themselves from thoughts and feelings by turning attention toward the “noticing self,” rather than sticking to difficult thoughts. (4) Identification of participant values and related behavioral goals will increase engagement in values-driven behavior, or committed action. Potential barriers to adopting values-driven behaviors and maintaining committed action in the long term will be explored and addressed through metaphors and experiential techniques.

### ACT-Based Intervention plus Activity Tracker WITH Feedback

#### Combining ACT and behavioral change

In the last experimental condition, obese individuals will follow the same rehabilitation program in the inpatient phase of the behavioral change condition, with the addition of four brief ACT sessions of duration 45-min for a total amount of 3 h of one-to-one therapy sessions, exactly as in the ACT condition. In the 16-week outpatient phase, each participant receives feedback from an activity tracker following the same stepped protocol, but messages and feedback are informed by an ACT therapist, including values-based goal setting, the defusion process for difficult thoughts, mindfulness cues, and a set of ACT-consistent metaphors and messages.

Combining ACT, which is a technology itself [50], with an app and wearable technologies is quite innovative. Some commercial apps are already available, but they are not developed in the Italian language.

### Recruitment of the study population, inclusion and exclusion criteria

Inpatients will be eligible when they will meet the following inclusion criteria at the admission to the hospital: (1) age between 18 and 70 years, (2) obesity according to the World Health Organization (WHO) criteria (body mass index (BMI) ≥ 30), (3) provision of written and informed consent to participate, (4) technology friendly to receive feedback through smartphone, e-mail, and wearable devices. Exclusion criteria for the study are the following: (1) other severe psychiatric disturbance diagnosed by the Diagnostic and Statistical Manual of Mental Disorders (DSM-5) that causes functional impairment or might compromise treatment adherence criteria, (2) severe visual difficulties, (3) important limitations of movement in particular subjects for which physical activity is not recommended, (4) concurrent medical condition not related to obesity. DSM-5 [87] will be used as a screening tool for psychiatric disorders. The Medical Ethics Committee of Istituto Auxologico Italiano approved the study protocol and the informed consent process.

### Randomization procedure

Using a double-blind procedure, we will randomly assign all of the participants to one of the four groups. The randomization scheme will be generated using the website Randomization.com (http://www.randomization.com). Randomization and follow-up assessments will be administered by independent practitioners blind to the research hypotheses and condition assignments. The G*Power software (v3.1.9.2) [35, 36] was used to compute the minimum sample size required to conduct this study. All of the participants will be assessed with the Psychological General Well-Being Index (PGWBI) [47, 62, 85] five times: (1) at admission to the clinic, (2) at discharge, (3) at 3 months follow-up, (4) at 6 months follow-up, and (5) at 12 months follow-up. Thus, the *experimental condition* is considered as a between-group variable, and *time* is considered as a within-subject variable. The a priori statistics (partial η^2^ and correlation between measures) were computed by examining previous longitudinal studies using the PGWBI in subjects with obesity [9, 65]. The a priori partial η^2^ was set to assume a value of 0.015 (small effect size) [26, 30], the type I error (α) rate was set at 0.05 (two-sided), the power (1 – β) was set at 0.95 [26], and the a priori correlation between repeated measures was set at 0.50. Finally, sphericity was assumed. G*Power showed that there is a 95% chance to correctly reject the null hypothesis of no significant effect with an overall sample of 168 participants, 42 subjects for each group.

### Assessment procedure

According to the behavioral framework, direct measures are preferred to psychometric inventory when possible. In any case, to deepen the understanding of mechanisms of change, psychometric measures were included. Participants will be asked to complete all study measures before randomization. Follow-up repeated measures assessment will be completed on all study participants 6 weeks later (post-treatment) and at 3, 6, and 12 months follow-up.

Demographic data such as gender, age, marital status, level of education, and employment status will be assessed in order to control for these potential confounders, checking also for biological indicators of risk—obesity, according to WHO criteria, with a BMI of 30 or more [67].

Primary outcome is physical activity as assessed continuously by wearable technology. Activity trackers will collect data, by direct measurement, of daily steps, physical activity, sleep, and food journal with the self-report app connected with the wearable device. Medical data and additional diagnoses (e.g., diabetes) that may confound results will be retrieved from clinical records.

Participants in both groups will provide a history of any psychological treatment or counseling to assess potential familiarity with mindfulness and acceptance-based therapies. A range of physiological and psychological outcome measures will assess the following: modifiable risk factors and recommended lifestyle dietary habits with the Mediterranean Diet Score [73], physical exercise with the International Physical Activity Questionnaire - Short Form (IPAQ-SF) [57] and the Acceptance and Action Questionnaire for Exercise (AAQ-EX) [85]. We also collect data about psychological well-being and quality of life (PGWBI) [47, 62, 85] and psychological flexibility (AAQ-II) [4, 49], a core process directly targeted by ACT interventions and involved as a mediator for well-being [53]. Adjusted change scores for all the outcome measures will be calculated in order to assess the ability of the interventions to produce changes over time.

### Psychotherapists and treatment fidelity

Motivational support and psycho-education will be supervised by experienced and certified psychotherapists from diverse backgrounds with specific training in CBT. The brief ACT intervention will be delivered by two psychotherapists with ACT and CBT background and formal training in ACT. They will receive monthly supervision by senior certified psychotherapists to ensure competent and uniform treatment delivery. A fidelity checklist, checked by a third, blinded therapist, will also be used for both treatments.

### Statistical analysis

As this study combines group design with different single-case design or, more in detail, randomly assigned multiple baseline design clustered by sub-sequential recruitment. Following methodological guidelines of RCT, the implementation of statistical analysis is linked to synergies that we can not previse at this point.

Statistical analyses will be performed using the R software [86]. Preliminary analyses will be carried out to assess the assumptions of parametric statistics; in case of violations, robust methods or data transformation will be used. Direct collected data (steps, weight, etc.) will be analyzed with an ITT approach with dropouts assumed to have regained 0.3 kg per month, an assumption already used in previous studies [16]. Dropouts will be excluded from the study.

The analysis of variance (ANOVA) will be used to examine between-group differences for all the primary and secondary outcomes at all time-points, focusing on the change in the scores. Corrected effect sizes (Hedges’ *g*) and 95% confidence interval (95% CI) will be calculated for both between-group and within-group differences.

Moreover, growth curve model analysis with a group variable (between conditions) will be used to evaluate the average growth from the baseline to 12 months after treatment for all of the outcome variables as performed in previous studies [52]. Moderation and meditational analysis will be implemented as more advanced statistical models. All data analyses will be performed using the Statistical Package for the Social Sciences (version 16.0; SPSS, Inc., Chicago, IL).

In addition, considering that the present study combines different single-case designs, more detailed randomly assigned multiple baseline designs clustered by sub-sequential recruitment, and a group design following methodological guidelines of RCTs, the implementation of statistical analysis is linked to synergies that we cannot previse at this point. For the single-case design, we use a multiple-baseline procedure with the same number of data points for all the subjects assigned to a cluster. In each multiple-baseline cluster we randomly assign the length of the baseline and, consequently, of the treatment. Detailed visual inspection and graphical analysis will be use, followed by a randomization test to ensure statistical power to the visual analysis [37, 38].

## Discussion

Due to the novelty of using a combination of behavioral change strategies with acceptance and mindfulness-based therapy with a wearable device to promote a healthful lifestyle in the post-rehabilitation period, no well-defined hypotheses have been established about the possible contribution of this kind of intervention in comparison with the “gold standard” multidisciplinary treatment chronic conditions linked to obesity. With the development of mobile technology and brief clinical protocols, new interventions in the field of obesity and connected chronic pathologies have to be evaluated.

The mACTonHEALTH study is quite innovative for different reasons. First, the combination of single-case designs with an RCT is uncommon, particularly with medium-scale studies. Second, the use of a wearable device activity tracker both as a device for data collection measuring the primary outcomes and as a device to provide contingent feedback, assumed as one of the two independent variables, is unique. Third, directly combining brief ACT intervention and behavior modification strategies with mobile-tech implementation is another element of innovation. Furthermore, although ACT is becoming well studied in the traditional clinical context, large-scale application to both medical and natural settings has not largely occurred. Including in the assessment procedure directly collectable data as indicators of both physical and psychosocial health is unique. Also, the inclusion of objective physiological outcome measures constitutes an aspect of innovation in ACT research. Last, the study will focus on mediation mechanisms underlying the treatment’s success and present possible links to systematic models on psychological flexibility, a core process assessed in ACT research.

The particular experimental design, using both directly measured outcomes and psychometric measures, particularly aimed at checking for mediation and moderation effects, is also one of the limits of the study. The complex experimental design with multiple independent variables requires a higher number of participants and could weaken the findings if the conclusions are not clear. Another limit of the study is represented by the absence of a real group without activity trackers. This decision is due to the importance of gathering data only collectable by the trackers, but even when the wearables collect data in “silent” mode, without providing any feedback, participants wear the devices. Even assuming that the effect of simply wearing a similar device is small, we cannot check directly. Despite these limitations and others of lesser importance, such as the limited number of psychometric inventories and the relatively small sample size of each subgroup, the benefits of this experimental design seem to overcome the downsides. First of all, direct measure of movement and physical activity represents an essential outcome, rarely collected in such a sample. Moreover, despite the downside of a small sample, a similar method could be used as an efficient way to test different hypotheses and to control for interactions between variables.

To the best of the researcher’s knowledge, this study will be the first RCT to examine the effectiveness of combining contingent feedback provided with wearable devices with ACT-based interventions to make the lifestyle more healthful according to medical and behavioral prescription among obese patients. This study is also one of the first examples of a large implementation of multiple single-case designs with a continuous data collection, made possible through the implementation of wearable technology. Conducted in the context of clinical practice, this trial will potentially offer empirical support to alternative interventions to improve radical long-time change in life habits and consequently promote quality of life, reducing mortality and morbidity rates among an obese population. Furthermore, by virtue of its short duration, the program could potentially also be utilized for health promotion in non-hospitalization contexts and primary or secondary prevention of obesity and linked conditions.

## Additional files

Additional file 1: The TIDieR (Template for Intervention Description and Replication) checklist*. (PDF 273 kb)

Additional file 2: CONSORT 2010 checklist of information to include when reporting a randomised trial*. (DOC 218 kb)

Additional file 3: SPIRIT 2013 checklist: recommended items to address in clinical trial protocol and related documents*. (PNG 601 kb)
