# Supplementary figures and images for: ACTonHEALTH study protocol: promoting psychological flexibility with activity tracker and mHealth tools to foster healthful lifestyle for obesity and other chronic health conditions
Source: Trials. 2018 Nov 29;19:659. doi: 10.1186/s13063-018-2968-x (PMC6262958; doi:10.1186/s13063-018-2968-x)

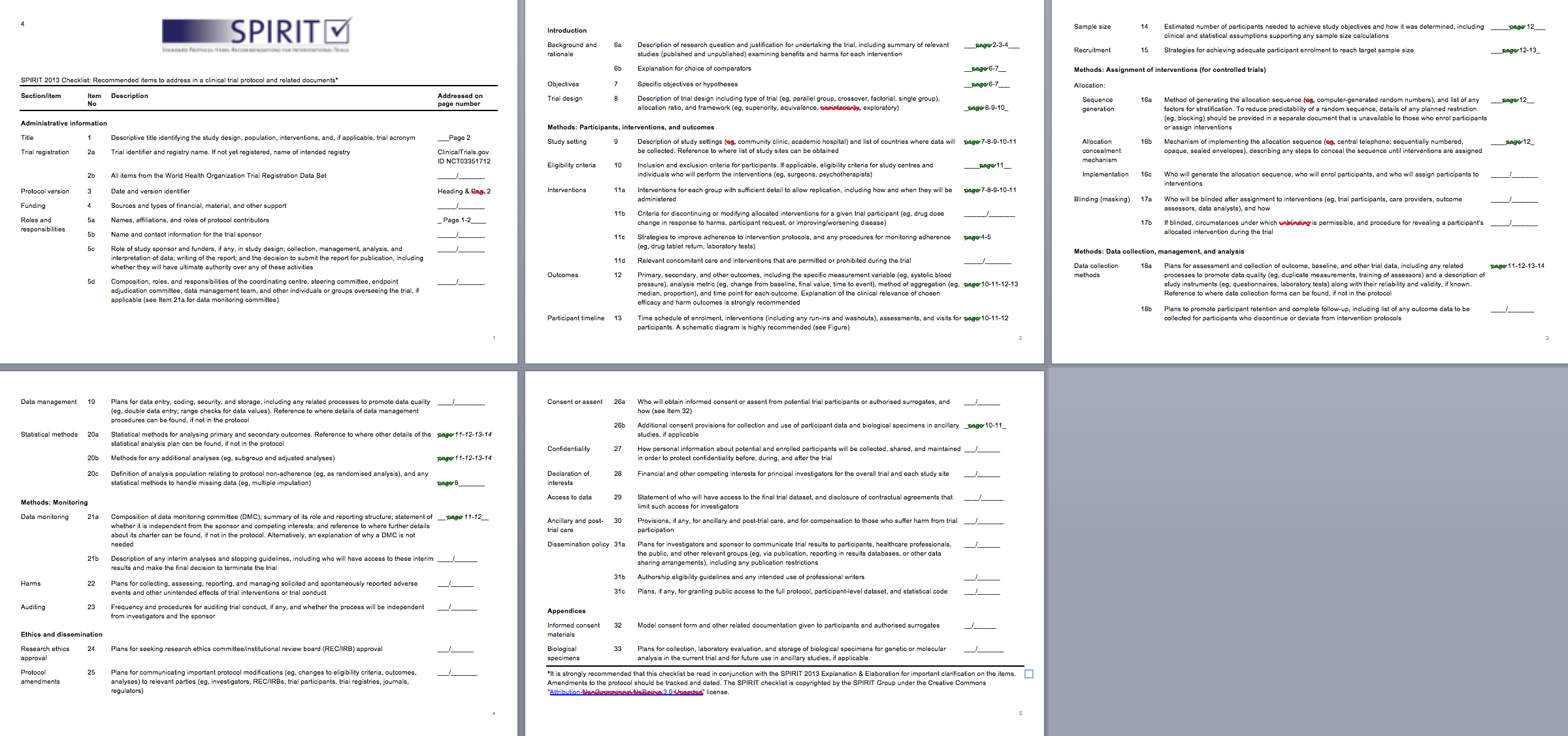

Supplement: Supplementary file 3 — SPIRIT 2013 checklist: recommended items to address in clinical trial protocol and related documents*. (PNG 601 kb) [file 13063_2018_2968_MOESM3_ESM.png]
